# Serum amyloid a, a potential biomarker both in serum and tissue, correlates with ovarian cancer progression

**DOI:** 10.1186/s13048-020-00669-w

**Published:** 2020-06-09

**Authors:** Ze Li, Yongwang Hou, Meng Zhao, Tianning Li, Yahui Liu, Jiao Chang, Li Ren

**Affiliations:** 1 Department of Laboratory, National Clinical Research Center for Cancer, Key Laboratory of Cancer Prevention and Therapy, Tianjin’s Clinical Research Center for Cancer, National Human Genetic Resources Sharing Service Platform, Tianjin Medical University Cancer Institute and Hospital, Tianjin, China; 2 Department of Laboratory, the First Affiliated Hospital of Hebei North University, Hebei, China; 3 School of Medical Laboratory, Tianjin Medical University, Tianjin, China

**Keywords:** Serum amyloid a, Ovarian Cancer, Matrix metalloproteinases, epithelial–Mesenchymal transition

## Abstract

**Background:**

Ovarian cancer is the most fatal gynecologic malignancy worldwide due to its vagueness, delay in diagnosis, recurrence, and drug resistance. Therefore, a new type of ovarian cancer treatment prediction biomarker is urgently needed to supplement existing tools. A total of 230 people participated in this study. Out of this figure, 100 participants were patients who underwent an ovarian tumor operation, another 100 participants were ovarian benign patients, and the remaining 30 participants were healthy women. Cancer (experimental) group were 100 patients who underwent ovarian tumor operation, while the control groups were 130 participants consisting of 100 ovarian benign patients and 30 healthy women. Levels of SAA, carbohydrate antigen-125 (CA-125), and human epididymis protein 4 (HE4) were assessed using standard laboratory protocols. A total of 5 ovarian cancer tissues and paracancerous tissues were collected and then stored at − 80 °C until the qRT-PCR assay was conducted.

**Results:**

The ROC curve of SAA concentration in ovarian cancer was plotted to obtain the area under the curve AUC = 0.889, the cut-off value 17.05 mg/L, the sensitivity 78.4% and specificity 86.5%. Compared with pretreatment, the level of serum SAA decreased significantly after treatment. The results revealed that there was a significant correlation between the level of serum SAA and advanced FIGO stage, histology subtype, lymphatic invasion, and distant metastasis (*p* = 0.003,0.002,0.000 and 0.001). The quantitative Reverse transcription polymerase chain reaction (qRT-PCR) assay revealed that the Messenger RNA (mRNA) of SAA-1 and SAA-4 was much higher in cancer tissues than in adjacent tissues, and MMPs was up-regulation including MMP-1, MMP-9 and MMP- 12 in OVCAR-3 cell stimulated by SAA. The transwell assay revealed that SAA could promote OVCAR-3 cell migration. Moreover, SAA can regulate EMT markers and promote AKT pathway activation.

**Conclusions:**

In summary, our results demonstrated that SAA may be a potential diagnosis and treatment prediction biomarker. The SAA promotes OVCAR-3 cell migration by regulating MMPs and EMT which may correlate with AKT pathway activation.

## Background

Ovarian cancer is a common clinically malignant tumor for women, and its morbidity has reached 6.31/10 million while the mortality rate was approximately 2.73/10 million [1]. Approximately, 60 to 70% of ovarian cancer patients have progressed to stage III-IV or have developed abdominal metastases because the patients’ early symptoms are often insignificant [2]. Presently, the 5-year survival rate for the disease is still as low as 30%, and the prognosis is poor despite the advancement in surgical techniques and methods of radiotherapy and chemotherapy for ovarian cancer [3]. Research has also shown that patients in early stage (FIGO I and II) have a better prognosis than those in advanced stage (FIGO III and IV) [4]. Therefore, early diagnosis of ovarian cancer is important for patients’ prognosis. Based on available reports, the serum markers of ovarian cancer mainly include CA-125 and HE4. However, CA-125 detection is poorly performed in the diagnosis of patients with early ovarian cancer [5]. The study has reported that the sensitivity and specificity of CA125 are 0.796 and 0.825 [6]. Although HE4 has greater specificity than CA125, the sensitivity has a varying result [6–8]. Therefore, it is important to look for a new biomarker in the serum that can help in diagnosing and predicting ovarian cancer. Previous study has revealed that acute phase serum amyloid A in ovarian cancer is an important component of Proteome diagnostic profiling [9]. In this study, we explored whether serum amyloid A could be a potential biomarker for ovarian cancer.

Serum amyloid A (SAA), an acute phase protein, is mainly synthesized in the liver, dramatically increasing during inflammatory diseases [10]. The level of serum SAA can elevate more than 1000 folds during inflammation [11, 12]. Therefore, SAA has been long considered as a sensitive marker of inflammation [13]. Convincing evidence has shown that chronic infection and inflammation especially bio-synthesis and secretion of pro-inflammatory cytokines is associated with cancer [14–16]. Moreover, it is reported that the concentration of SAA is significantly high in different types of cancer including lung cancer [17], breast cancer [18, 19], uterine cervical cancer [20], renal cancer [21], gastric cancer [22], and others [23, 24].

In this study, we investigated the expression of SAA in ovarian tumor tissue and normal tissue and assessed the relationship between SAA and prognosis of ovarian cancer patients. Furthermore, we provided the advantages of SAA in diagnosing ovarian cancer combinations of CA125 and HE4 and found that SAA could be a potential biomarker. In addition, we explained potentially that SAA promotes OVCAR-3 migration by inducing MMPs expression.

## Results

### Serum amyloid a (SAA) is overexpressed in ovarian cancer

**Fig. 1:**
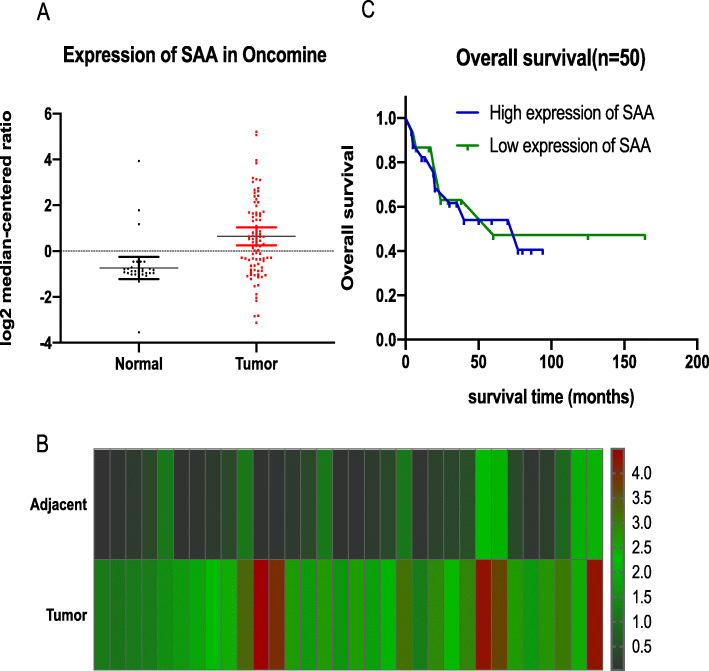
SAA is overexpressed in ovarian cancer. **a.** SAA is overexpression in ovarian cancer samples (109 cases) compared with adjacent normal tissue samples (37 cases) in Oncomine profiles, *P* = 0.008, Wilcoxon-Signed rank test. **b.** SAA expression increased significantly in 32 paired ovarian tumor tissues (Tumor) and their adjunct normal tissues (Adjacent) in Oncomine profiles. Data are presented as the Mean ± SD, Adjacent (0.575 ± 0.698), Tumor (2.450 ± 0.982). **C.** Kaplan-Meier survival curves demonstrated that the overall survival of patients with high expression of SAA was not significantly different from those with low SAA expression in the same profiles, *P* = 0.8533, Log-rank (Mantel-Cox) test

Through analysis of SAA expression in Oncomine datasets profiles from ovarian cancer patients, we found that it is overexpression in ovarian cancer samples (109 cases) compared with adjacent normal tissue samples (37 cases) (*P* = 0.008, Wilcoxon-Signed rank test) (Fig. 1a). We further analyzed the expression of SAA in a total of 37 paired ovarian tumor tissues in these datasets and found that it was significantly upregulated in 32 of the ovarian tumor tissues compared with their adjacent normal tissues. Data were presented as the Mean ± SD, Adjacent (0.575 ± 0.698), Tumor (2.450 ± 0.982). (Fig. 1b). Also, in this Oncomine datasets, Kaplan-Meier survival curves demonstrated that the overall survival of patients with high expression of SAA was not significantly different from those with low SAA expression in the same profiles; *P* = 0.8533, Log-rank (Mantel-Cox) test. (Fig. 1c).

### Overexpression of SAA is associated with advanced clinical features in ovarian cancer

A total of 100 patients from the entire cohort were included for analyses. Details of patients’ characteristics are shown in Table 1. We further analyzed the association between SAA and the clinicopathological characteristics of ovarian cancer. There was no significant association between SAA expression and age, and pathological differentiation and tumor site. The results of the logistic regression analysis revealed that there was a significant correlation between the level of serum SAA and advanced FIGO stage, histology subtypes, lymphatic invasion, and distant metastasis (*p* = 0.003,0.002,0.000 and 0.001) (Tables 1 & 2).

**Table 1:** Univariate analysis for the association of various clinicopathologica features with SAA expressions of patients with ovarian cancer

Feature	N.(*n* = 100)	SAA [mg/L, M (QR)]	Z	P
Age(y)				
≥ 60	55 (55%)	51.7 (15.63,187.95)	1.254	0.516
< 60	45 (45%)	57.6 (18.35,180.7)		
FIGO stage				
I-II	30 (30%)	8.8 (6.30,32. 3)	7.106	0.003
III	25 (25%)	42.1 (12.8166.35)		
IV	45 (45%)	68.30 (22.63,187.95)		
Histology subtype				
Serous	64 (64%)	8.8 (6.4,79.8)	−3.017	0.002
Endometrioid	36 (36%)	55.8 (22.85,192.65)		
Lymphatic invasion				
No	41 (41%)	17.7 (7.42,43.47)	−3.927	0.000
Yes	59 (59%)	157.4 (66.43,216.6)		
Distant metastasis				
NO	55 (55%)	13.60 (6.15,46.23)	−3.416	0.001
Yes	45 (45%)	80.90 (29.23,211.03)		
ER				
Positive	70 (70%)	27.90 (8.20,156.30)	0.810	0.418
Negative	30 (30%)	17.40 (6.90,108.40)		
PR				
Positive	39 (39%)	32.30 (6.85,136.60)	0.265	0.791
Negative	61 (61%)	27.30 (7.70,136.00)

**Table 2:** Multivariate Logistic regression analysis for the association of clinicopathologica features with SAA expressions of patients with ovarian cancer

Feature	B	S.E	Wald	df	Sig.	Exp(B)	95% CI	
FIGO stage							Lower	Upper
I-II	1.156	0.598	6.813	1	0.009	4.758	1.475	15.350
III	2.457	0.871	7.955	1	0.004	11.667	2.116	64.326
IV	2.799	1.008	7.715	1	0.005	16.430	2.279	118.424
Histology subtype	2.868	1.172	5.547	1	0.013	17.636	1.773	150.854
Lymphatic invasion	2.890	1.219	5.622	1	0.018	18.000	1.650	196.309
Distant metastasis	3.632	1.166	9.710	1	0.002	37.800	3.849	371.271

### Serum SAA levels in patients with ovarian cancer after treatment

**Fig. 2:**
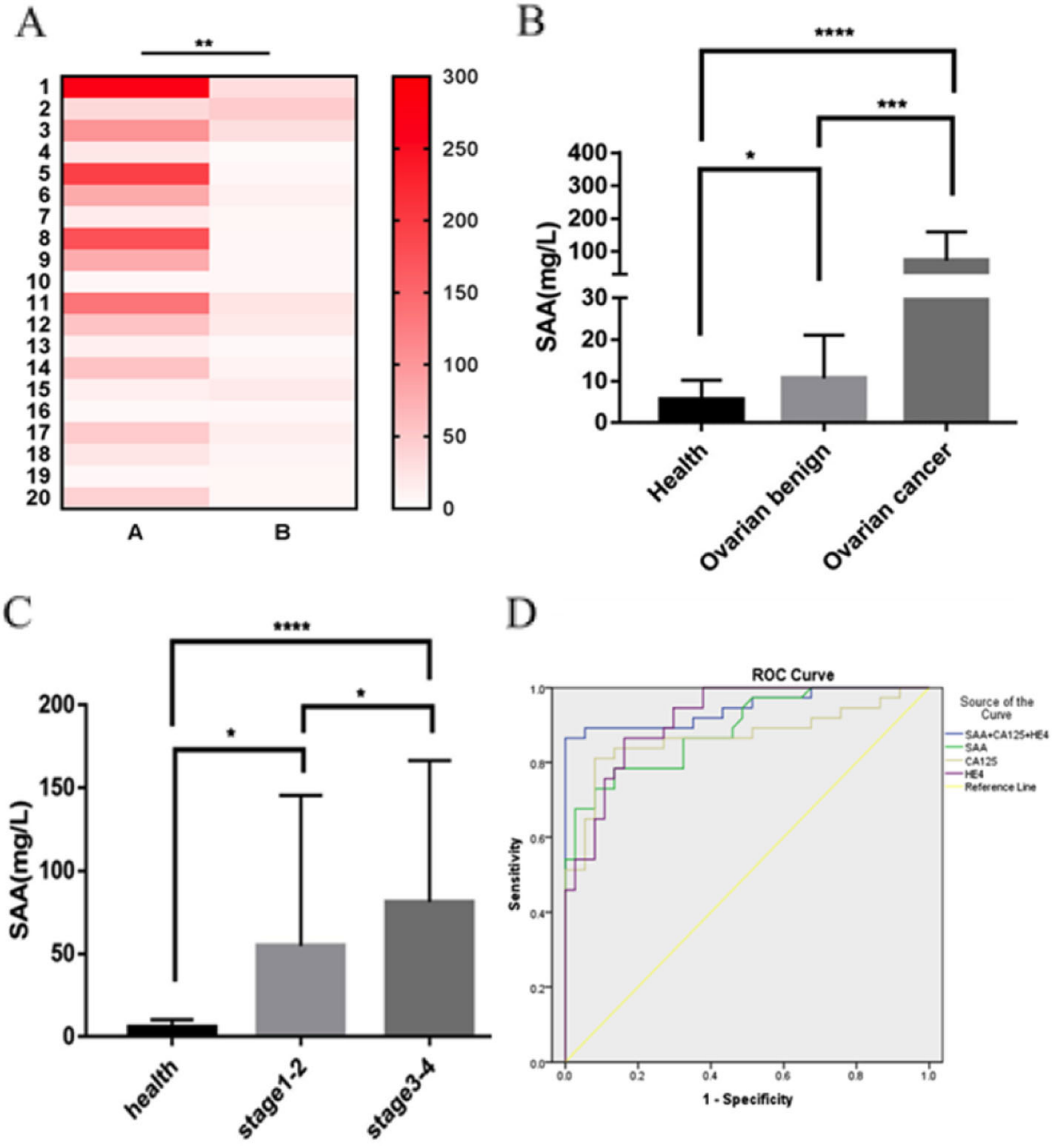
SAA as a prognostic biomarker in ovarian cancer. **a.** Heat-map of SAA between pretreatment (A) and post-treatment(B). *n* = 20, ** *P* < 0.01. **b.** Comparison of SAA expression in serum among healthy control group, ovary benign and cancer group, *P* = 0.189,0.000,0.000 **c.** High-FIGO stage patients (III + IV), SAA expression in serum was significant difference with healthy group, *p* = 0.0001. Low-FIGO stage patients(I + II), SAA expression in serum was difference with healthy group, *p* = 0. 0162. Between high-FIGO stage patients (III + IV) and low-FIGO stage patients(I + II), SAA expression in serum was difference, *p* = 0.0232. **d.** ROC curve of sensitivity versus specificity of SAA, CA125, HE4, and combinations of three makers. In combined detection of SAA, CA125, and HE4, AUC = 0.945, Sensitivity = 0.892, Specificity =0.973. Kruskal–Wallis /one-way ANOVA tests were used to test the statistical significant (B,C). (* *P* < 0.05, *** *P* < 0.001, **** *P* < 0.0001)

To estimate the effect of SAA in the treatment of ovarian cancer, we collected 20 patients’ serum after treatment because others failed to follow-up the treatment. Strikingly, compared with pretreatment, the level of serum SAA decreased significantly after treatment (Fig. 2a). Clinically, the result shows that doctors can assess the effect of treatment for ovarian cancer patients by detecting the level of serum SAA.

### SAA as a prognostic biomarker in ovarian cancer

We examined the concentration of serum SAA, CA125, and HE4 in health, ovarian benign Disease, and ovarian cancer, as shown in Table 3. Compared with the healthy group, the concentration of SAA in the ovarian cancer group was significantly higher and the difference was statistically significant (*P* = 0.000). Also, there was a significant difference between benign ovarian disease group and ovarian cancer group (*P* = 0.000) (Fig. 2b.). There was significant associated between advanced FIGO stage. Compared with the healthy group, the concentration of SAA in FIGO III-IV group was significantly higher and the difference was statistically significant (*P* = 0.000). In addition, the FIGO I-II group was different from the healthy group ((*P* = 0.016) (Fig. 2c). Kruskal–Wallis /one-way ANOVA tests were used to test the statistical significant.

**Table 3:** The median serum SAA, CA125 and HE4 concentration

Group	N	SAA [mg/L,M (QR)]	CA125 [U/L, M (QR)]	HE4 [pmol/L,M (QR)]
Health	30	4.95 (3.27–7.67)	17.79 (10.48–30.77)	21.39 (14.38–30.71)
Benign disease	36	6.10 (4.70–9.45)	35.86 (15.82–53.16)	47.73 (37.39–74.64)
Ovarian cancer	36	32.30 (11.20–119.6)	640.00 (251.65–1218.50)	259.5 (125.00- 736.00)

The ROC curve of SAA, CA125, and HE4 was shown (Fig. 2d). As shown in Table 4, the ROC curve of SAA concentration in ovarian cancer was plotting to obtain the area under the curve AUC = 0.889, the cut-off value is 17.05 mg/L, sensitivity is 78.4%, and specificity is 86.5%. The ROC curve for the diagnosis of ovarian cancer with CA125 concentration was plotted. The AUC was 0.868; the cut-off value was 245.30 mg/L; the sensitivity was 81.1%; the specificity was 91.9%. The ROC curve of HE4 for diagnosis of ovarian cancer showed that AUC = 0.917, cut-off value at this time was 98.01 mg/L, sensitivity was 86.5%, and specificity was 83.8%. While in combined detection of SAA, CA125, and HE4, the AUC was 0.945, the sensitivity was 89.2%, and the specificity was 97.3%. These data suggest that SAA can be used as a potential biomarker, and the combined detection of SAA, CA125, and HE4 shows a good value for the diagnosis of ovarian cancer.

**Table 4:** Receiver operating characteristic (ROC) curve of the diagnostic power of serum SAA level for ovarian cancer

	cut-off	AUC	YOUDEN	Sensitivity	Specificity	95% CI	
						Lower	Upper
SAA (mg/L)	17.05	0.889	0.649	0.784	0.865	0.861	0.961
CA125(U/L)	245.30	0.868	0.730	0.811	0.919	0.778	0.951
HE4(pmol/L)	98.01	0.917	0.703	0.865	0.838	0.857	0.977
Combined	–	0.945	0.838	0.892	0.973	0.891	0.999

**Fig. 3:**
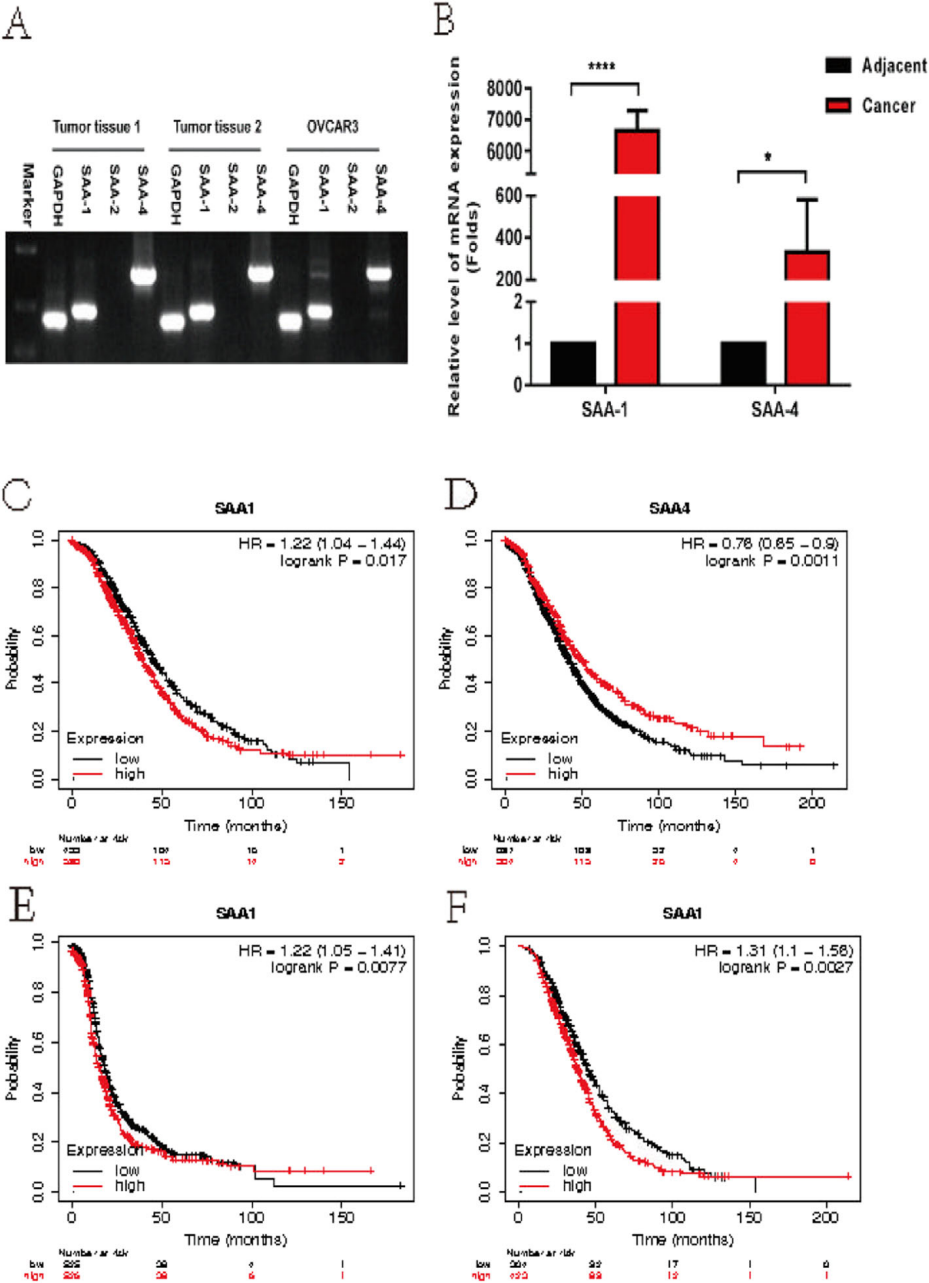
SAA modulates the migration of ovarian cancer cells. **a.** Expression of SAA-1, SAA-2, and SAA-4 in ovarian tumor tissues and OVCAR3 cell. **b.** cDNA from 5 ovarian cancer tissues and paracancerous tissues were analyzed for SAA-1 and SAA-4 levels by qRT-PCR. (**C-F**). Survival analysis by using Kaplan Meier (KM) plotter online tool. Survival curves in all patients according to SAA-1(**c**) and SAA-4 (**d**) expression in ovarian cancer. (**E, F**). Survival curves in patients with different histology subtypes according to the SAA expression. **e.** OS in Serous patients **f.** OS in Endometrioid patients. Kaplan-Meier survival curves were generated and compared statistically by the log-rank test. *P* < 0.05 (* *P* < 0.05, *** *P* < 0.001, **** *P* < 0.0001)

Kaplan-Meier survival curves showed that patients with high SAA1 expression had worse OS than with those low SAA1, but high SAA4 expression had better OS than those with low SAA4 (Fig. 3c, d). In addition, when the analysis was confined to histology subtypes, the serous group and the endometrioid group have a significant difference in OS based on the SAA level (Fig. 3e, f).

### SAA modulates migration of ovarian cancer cells

**Fig. 4:**
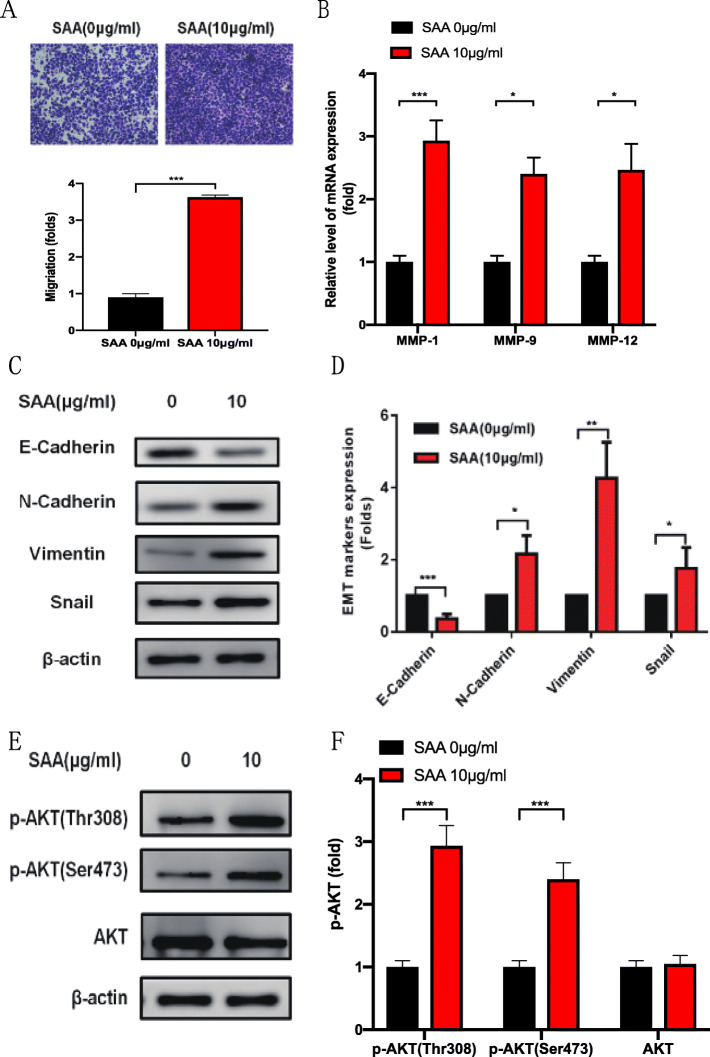
AKT signaling pathway is regulated by SAA. **a.** OVCAR-3 cell was treated with SAA 0 μg/ml and 10 μg/ml 12 h respectively, with representative graphs of OVCAR-3 cells in migration. The data were shown as the means±SD (*n* = 3). The fold change is 2.46, *P* = 0.0345, t-test. **b.** The mRNA of MMP-1, MMP-9, and MMP-12 were assayed after OVCAR-3 cell had been treated with SAA 0 μg/ml and 10 μg/ml 24 h respectively. The data were shown as the means±SD (*n* = 3). The fold changes respectively are 2.3,1.7,2.0. *P* = 0.0001,0.0137,0.0224. (**c, d**) RT-qPCR and Western blot analysis of EMT markers in OVCAR-3 cell treated with SAA 0 μg/ml and 10 μg/ml 24 h respectively. The data were shown as the means±SD (*n* = 3). The fold changes respectively are 0.35,2.1,3.5,1.6. *P* = 0.0008,0.0362,0.0013. (**e, f**)**.** Western blot analysis of phosphorylation AKT in OVCAR-3 cell treated with SAA 0 μg/ml and 10 μg/ml 24 h. The fold changes respectively are 2.18,1.97,0.01. *P* = 0.0004,0.0006,0.2148. Statistical significance was calculated used t-test(A) and Wilcoxon Signed Rank test with one unpaired t-test per row(B-F). (* *P* < 0.05, *** *P* < 0.001, **** *P* < 0.0001)

By determining SAA expression via gene set enrichment analysis (GSEA) [25, 26] and the Cancer Genome Atlas profiles, we found that SAA levels were positively correlated with the proliferation by affecting genes in cell cycle regulation. Firstly, we examined the expression of SAA-1, SAA-2, and SAA-4 in ovarian tumor tissues and OVCAR-3 cells. We found that SAA-1 and SAA-4 could be expressed in ovarian tumor tissues and OVCAR-3 cell, but SAA-2 could not (Fig. 3a). These results were consistent with previous studies [27]. Then, we examined the mRNA expression of SAA in ovarian cancer tissues and adjacent tissues. We found that the mRNA of SAA-1 and SAA-4 was much higher in cancer tissues than in adjacent tissues by qRT-PCR (Fig. 3b). The migration assays revealed that overexpression of SAA significantly increased the OVCAR-3 cell numbers, which were approximately 2.46-fold higher at day 5 after treated with 10 μg/ml SAA compared to 0 μg/ml SAA (Fig. 4a), thus, we focused on the MMPs. As expected, after treating OVCAR-3 cell with 10 μg/ml SAA 24 h, the mRNA expression of MMP-1, MMP-9, and MMP-12 (Fig. 4b) were lower in the control group. Our results demonstrate that SAA did not only induce MMP-9 but also induces the expression of MMP-1 and MMP-12.

### AKT signaling pathway is regulated by SAA

We further explored the role of SAA in metastasis. Epithelial–mesenchymal transition (EMT) enables tumor cells to gain invasion and migration capabilities [28], and this phenomenon has been reported in different malignancies. Both qPCR (Fig. 4c) and western (Fig. 4d) results show that SAA can prompt mesenchymal markers N-cadherin, Vimentin, and snail up-regulation and suppress epithelial marker E-cadherin expression. However, the regulation of EMT by SAA is not clear. Because AKT pathway plays a very important role in EMT [29], we explored the influence of SAA on AKT pathway. Our results demonstrate that SAA can induce the phosphorylation of AKT on Ser473 and Thr308, although the total AKT did not change (Fig. 4e, f). These data suggest that SAA promotes OVCAR-3 cell migration by stimulating MMPs up-regulation and promoting EMT which may correlate with AKT pathway activation.

## Discussion

We observed that in combined detection of SAA, CA125, and HE4, the AUC was 0.945, the sensitivity was 89.2%, and the specificity was 97.3% (Fig. 2d). These data suggest that SAA can be used as a potential biomarker, and combined detection of SAA, CA125, and HE4 shows a good value for the diagnosis of ovarian cancer, which has been not reported. But our analysis lacked SAA data about relapsed patients. Our analyses of the relationships between SAA expression and clinicopathological features showed that SAA was significantly associated with advanced FIGO stage, histology subtypes, lymphatic invasion, and distant metastasis (Table 2). Among these significant factors, we also conducted a stratified study on the correlation between SAA and histology subtypes. Then we observed that there was a significant difference between the serous group and the mucinous group, which has been previously reported. Subsequently, we conducted a multivariate logistic analysis, which revealed that clinical features such as advanced FIGO stage, histology subtypes, lymphatic invasion, and distant metastasis were significantly and independently associated with high SAA expression (Table 3).

More importantly, Kaplan-Meier survival curves showed that patients with high SAA1 expression had worse OS than with those low SAA1, but high SAA4 expression had better OS than those with low SAA4 (Fig. 3c, d). In addition, when the analysis was confined to histology subtypes, the serous group and the endometrioid group have a significant difference in OS according to the SAA level (Fig. 3e, f). These findings indicate that high SAA expression affects survival mainly in different histology subtypes, which is consistent with our finding that SAA expression was significantly and independently associated with histology subtypes. In clinical practice, the prediction of tumor progression after treatment is of great importance. The ideal prognostic marker can provide a basis for evaluating the clinical outcome, which helps clinicians in choosing the best treatment strategy for patients to avoid overtreatment or undertreatment. In this study, the level of serum SAA decreased significantly after treatment when compared with pretreatment. (Fig. 2a). This could be an ideal predictive biomarker for treatment outcome and a reasonable therapeutic target in epithelial ovarian cancer.

We demonstrated that SAA was upregulated in ovarian cancer cells and tissues (Fig. 3a, b) (Fig. 4a). SAA also induced matrix metalloproteinases (MMPs), includes MMP-9, MMP-1, and MMP-12 (Fig. 4b-d). O’Hara R, and Tamamoto T et al. had reported that SAA was involved in adhesion, migration, and tissue infiltration of inflammatory cells, induced matrix metalloproteinases which could interact with degrading extracellular matrix (ECM) controlling the diffusion and migration of cells [30–35]. Possible mechanisms of SAA for stimulating MMP-9 might be via formyl peptide receptor like-1-mediated signaling [36, 37]. Ren Y and Liao WS studies have proved that SAA could influence carcinogenesis by activating the transcription factor and nuclear factor kappa-B (NF-κB) [38, 39]. SAA may also favor tumor development by limiting immune anti-tumor by stimulating the growth of regulatory T cells in a process involving IL-1β and IL-6 induction in monocyte [40]. Jae W. Lee, Meredith L. Stone and et al. reported that inflammatory responses mounted by hepatocytes are critical to liver metastasis in pancreatic ductal adenocarcinoma. Mechanistically, hepatocytes orchestrate this process through activation of IL-6/STAT3/SAA signaling, which alters the immune and fibrotic microenvironment of the liver to establish a pro-metastatic niche [41]. Thus, we consider that SAA is involved in neoplastic progress and with immunosuppression and promotion of metastatic niches.

We found that SAA promotes OVCAR-3 cell migration by stimulating MMPs up-regulation and promoting EMT, which may correlate with AKT pathway activation. AKT is a significant mediator of the cell cycle usually highly activated by its phosphorylation at both the Thr308 and Ser473 sites. In human cancers, which promotes cancer cell proliferation and migration, as well as provides resistance against apoptosis [42, 43]. More importantly, p-AKT is a crucial modulator of glucose metabolism in different cells [44]. However, further study is needed on the specific mechanism of SAA’s regulation of AKT pathway activation.

Our study has the following limitations. First, the sample size of the analyses was relatively small. Second, prospective studies are required to verify our findings. Third, the strengthening of our hypothesis by basic study data was lacking.

## Conclusions

Based on the current findings, we assume that SAA is involved in neoplastic progress. In conclusion, SAA may serve as a useful biomarker for poor prognosis. Clinical diagnosis combine with SAA expression may help to assess therapeutic outcomes.

## Methods

### Patients and samples

Blood samples were collected using a standardized procedure. After obtaining patients’ approval, serum samples were collected before the initial surgery from 200 patients comprising samples from 100 patients with average age 58.81 ± 8.7 years with ovarian cancer and 100 patients with average age 53.78 ± 16.69 years with ovarian benign disease. All patients were hospitalized at Tianjin Medical University Cancer Hospital in China between 2017 and 2019. Patients with rheumatoid arthritis, acute inflammations infection were excluded from this study. Samples from 30 healthy age-matched women with average age 56.07 ± 13.93 years were used as the normal controls. The results of one-way ANOVA showed that there was no statistical significant difference between the three groups of subjects (*P* = 0.395). Venous blood samples were collected in pyrogen-free tubes, allowed to clot at 4 °C for 1 h, and then centrifuged at 2000×g for 10 min. The upper serum layers were carefully obtained and divided into separate vials, and stored at − 20 °C until the assay was conducted. Five ovarian cancer tissues and paracancerous tissues were collected and then stored at − 80 °C until the assay was conducted.

### Latex enhanced immunoturbidimetric assay of SAA

The serum level of SAA was determined using a commercial Kit for Serum amyloid A protein assay (Ningbo Purebio Biotechnology Co, Ltd) and Automatic Analyzer H7180ID following the manufacturer’s instructions. The instruction states that if the concentration of the sample exceeds the linear range, it should be diluted with normal saline and re-measured.

### Cell cultures

Ovarian cancer cell lines, OVCAR-3 were obtained from the Type Culture Collection of Chinese Academy of Sciences, and the culture was maintained according to their recommendations.

### In vitro migration assay

For the transwell migration assay, OVCAR-3 cells (2 × 105/well) in 200 μl of serum-free media (RPMI DMEM) were placed in the upper chamber (Corning, Cambridge, USA) of each insert with Apo-SAA (Sigma-Aldrich, USA) (0, 5and 10 μg/ml). Thereafter, medium supplemented with 20% fetal bovine serum (600 μl) was added to the lower chambers. After 12 h of incubation at 37 °C, the upper surface of the membrane was wiped with a cotton tip, and the cells attached to the lower surface were stained for 15 min with crystal violet. The assays were performed in triplicate.

### RNA isolation and qRT-PCR assay

Total RNA was isolated from fresh tissue samples using the Total RNA Extraction Kit (Solarbio Science Technology CO. Ltd., Beijing, China) following the manufacturer’s instructions. Total RNA (2 μg) was used for the synthesis of the first strand cDNA using the Revert Aid First Strand cDNA Synthesis Kit (Thermo Fisher, USA) under the conditions recommended by the supplier. For qRT-PCR, the SYBR green mix (Applied Biosystems) was used to run on PCR on a LightCycler 96 System (Roche, Germany). The data were displayed as 2-∆∆Ct values with GAPDH as the control. Sequences of the RT-PCR primers were shown in supplemental Table 1.

### Protein extraction and western blot analysis

Briefly, for tissue protein extraction, the OVCAR3 cells were homogenized in ice-cold RIPA (radioimmunoprecipitation assay) lysis buffer. The cell lysates were incubated in ice for 30 min followed by centrifugation at 12, 000 rpm for 10 min. The protein concentration of cells extracts was determined using the BCA Protein assay Kit (Pierce). For western blot, the protein (20 μg) were loaded into 12% polyacrylamide-SDS gradient gels and then transferred to a PVDF membrane. The PVDF membrane was blocked with 1% BSA. The antibodies E-cadherin (Cell Signaling), Vimentin (Cell Signaling), snail (Cell Signaling), N-cadherin (Cell Signaling), β-actin (Sigma-Aldrich), total AKT (Cell Signaling), p-AKT (Ser473) (Cell Signaling), p-AKT (Thr308) (Cell Signaling), were applied for protein detection.

### Data analysis

Kaplan Meier (KM) plotter (http://kmplot.com/analysis) online tool to associate the presence of genomic transcripts with overall survival (OS) in ovarian cancer patients.

### Statistical analysis

The normality of the variables was tested by the Shapiro-Wilk normality test. Wilcoxon-Signed Rank test was used in comparing the differences between the continuous variables. Kruskal–Wallis tests and Mann-Whitney U were used to compare the differences among more than two groups. Correlation coefficients were computed using Partial Spearman and distance correlation analyses. Logistic regression was used to test the univariate and multivariate analyses of the clinicopathologic factors associated with SAA. Kaplan-Meier survival curves were generated and compared statistically by the log-rank test. The *p*-value < 0.05 indicates a statistically significant. All statistical analyses were performed using IBM SPSS 22.0 and GraphPad Prism 8.

## Supplementary information

**Additional file 1: Table S1.** The primer sequences for real-time quantitative polymerase chain reaction (RT-qPCR)

## Data Availability

The data used to support the findings of this study are available from the corresponding author upon request.

## Abbreviations

SAA: Serum amyloid A
CA125: Carbohydrate antigen 125
HE4: Human epididymis secretes protein
ROC: Receiver operating characteristic curve
AUC: Area under curve
FIGO: International federation of gynecology and obstetrics
OS: Overall survival
MMP: Matrix metalloproteinase
EMT: Epithelial mesenchymal transition
NF-κB: Nuclear factor kappa-B
ECM: Extracellular matrix
