# Supplementary material for: Serum amyloid a, a potential biomarker both in serum and tissue, correlates with ovarian cancer progression
Source: J Ovarian Res. 2020 Jun 9;13:67. doi: 10.1186/s13048-020-00669-w (PMC7285470; doi:10.1186/s13048-020-00669-w)
Supplement: Supplementary file 1 — Additional file 1: Table S1. The primer sequences for real-time quantitative polymerase chain reaction (RT-qPCR) [file 13048_2020_669_MOESM1_ESM.docx]

**Table S1:** The primer sequences for real-time quantitative polymerase chain reaction (RT-qPCR)

| GAPDH | Forward  Reverse | 5’-GGAGCGAGATCCCTCCAAAAT-3’  5’-GGCTGTTGTCATACTTCTCATGG-3’ |
| --- | --- | --- |
| SAA-1 | Forward  Reverse | 5’-CTGCAGAAGTGATCAGCG-3’  5’-ATTGTGTACCCTCTCCCC-3’ |
| SAA-2 | Forward  Reverse | 5’-CTGCAGAAGTGATCAGCA-3’  5’-ATTATATGCATTATCTCAGC-3’ |
| SAA-4 | Forward  Reverse | 5’-CCAGTGAAAGCTGGCGTTCG-3’  5’-GAGAAGTGTGTGGCTCACAGCC-3’ |
| MMP-1 | Forward  Reverse | 5’-AAAATTACACGCCAGATTTGCC-3’  5’-GGTGTGACATTACTCCAGAGTTG-3’ |
| MMP-9 | Forward  Reverse | 5’-TGTACCGCTATGGTTACACTCG-3’  5’-GGCAGGGACAGTTGCTTCT-3’ |
| MMP-12 | Forward  Reverse | 5’-CATGAACCGTGAGGATGTTGA-3’  5’-GCATGGGCTAGGATTCCACC-3’ |
| E-cadherin | Forward  Reverse | 5’-CGAGAGCTACACGTTCACGG-3’  5’-GGGTGTCGAGGGAAAAATAGG-3’ |
| N- cadherin | Forward  Reverse | 5’-TCAGGCGTCTGTAGAGGCTT-3’  5’-ATGCACATCCTTCGATAAGACTG-3’ |
| Vimentin | Forward  Reverse | 5’-GACGCCATCAACACCGAGTT-3’  5’-CTTTGTCGTTGGTTAGCTGGT-3’ |
| Snail | Forward  Reverse | 5’-TCGGAAGCCTAACTACAGCGA-3’  5’-AGATGAGCATTGGCAGCGAG-3’ |
